# Effectiveness of a School-Based Physical Activity Intervention on Cognitive Performance in Danish Adolescents: LCoMotion—Learning, Cognition and Motion – A Cluster Randomized Controlled Trial

**DOI:** 10.1371/journal.pone.0158087

**Published:** 2016-06-24

**Authors:** Jakob Tarp, Sidsel Louise Domazet, Karsten Froberg, Charles H. Hillman, Lars Bo Andersen, Anna Bugge

**Affiliations:** 1 Department of Sport Science and Clinical Biomechanics, Research Unit for Exercise Epidemiology, Centre of Research in Childhood Health, University of Southern Denmark, Campusvej 55, 5230 Odense M, Denmark; 2 Department of Kinesiology and Community Health, University of Illinois at Urbana-Champaign, 906 South Goodwin Avenue, Urbana-Champaign, Illinois, 61801, United States of America; 3 Department of Sports Medicine, Norwegian School of Sport Sciences, Oslo 0806, Norway; Erasmus University Rotterdam, NETHERLANDS

## Abstract

**Background:**

Physical activity is associated not only with health-related parameters, but also with cognitive and academic performance. However, no large scale school-based physical activity interventions have investigated effects on cognitive performance in adolescents. The aim of this study was to describe the effectiveness of a school-based physical activity intervention in enhancing cognitive performance in 12–14 years old adolescents.

**Methods:**

A 20 week cluster randomized controlled trial was conducted including seven intervention and seven control schools. A total of 632 students (mean (SD) age: 12.9 (0.6) years) completed the trial with baseline and follow-up data on primary or secondary outcomes (74% of randomized subjects). The intervention targeted physical activity during academic subjects, recess, school transportation and leisure-time. Cognitive performance was assessed using an executive functions test of inhibition (flanker task) with the primary outcomes being accuracy and reaction time on congruent and incongruent trials. Secondary outcomes included mathematics performance, physical activity levels, body-mass index, waist-circumference and cardiorespiratory fitness.

**Results:**

No significant difference in change, comparing the intervention group to the control group, was observed on the primary outcomes (p’s>0.05) or mathematics skills (p>0.05). An intervention effect was found for cardiorespiratory fitness in girls (21 meters (95% CI: 4.4–38.6) and body-mass index in boys (-0.22 kg/m^2^ (95% CI: -0.39–0.05). Contrary to our predictions, a significantly larger change in interference control for reaction time was found in favor of the control group (5.0 milliseconds (95% CI: 0–9). Baseline to mid-intervention changes in physical activity levels did not differ significantly between groups (all p’s>0.05).

**Conclusions:**

No evidence was found for effectiveness of a 20-week multi-faceted school-based physical activity intervention for enhancing executive functioning or mathematics skills compared to a control group, but low implementation fidelity precludes interpretation of the causal relationship.

**Trial Registration:**

www.ClinicalTrials.gov NCT02012881

## Introduction

In recent years the attention given towards the role of physical activity on the scholastic and cognitive potential of children has increased [1–5]. Continuing to gather causal data regarding this relationship is an important argument for prioritizing physical activity in the school curriculum with potential implications for public health [6].

Cardiorespiratory fitness and measures of body fat have been associated with specific aspects of cognition known as executive functions in children [5] and adolescents [7]. Since executive functions are important correlates of academic achievement [8], this establishes a mechanism explaining better scholastic performance of physically active children [9], but physical activity in itself may also confer benefits [10]. Executive functions is an umbrella term related to top-down mental processes involved in the planning and selection of strategies that organize goal-directed actions [8]. Executive functions are comprised of working memory, cognitive flexibility and inhibitory control. Available evidence suggests a selective relationship between tasks requiring greater amounts of inhibitory control (involved in suppression of prepotent responses [11] and selective attention [8]) with cardiorespiratory fitness [5]. Until recently, this evidence was cross-sectional only [12], however prospective studies supporting the same role of cardiorespiratory fitness are now emerging [13]. Further, two randomized trials have now demonstrated that manipulation of physical activity can positively influence executive functions [14, 15] and academic performance [14]. While these results are encouraging, these trials were conducted as afterschool programs and included only children who were overweight [14] or included ≈ 50% overweigh children [15]. Hence, these trials do not provide evidence for the effect of daily physical activity in children with a normal-for-age body mass index (BMI) or, importantly, do not provide evidence for larger reaching initiatives such as those occurring within schools and during the school day. Also, both trials included pre-pubertal children whose cognitive response to manipulation of physical activity may be differ from adolescents in the midst of puberty.

The most recent systematic review on the association between physical activity and school performance, including only prospective observational or intervention studies, concluded that there was strong evidence for a positive prospective relationship [4]. The authors, however, emphasized that their conclusion was based on a limited number of adequately performed studies and few intervention studies. Furthermore, only one study had used an objective measure of physical activity, but only in a sub-sample [16]. Additional support for this notion is given in a recent non-randomized intervention study that used eight years of routinely collected academic performance data to show a higher proportion of students achieving Swedish national learning goals following a doubled provision of physical education [17]. Collectively, there is a need for more experimental research including objective assessment of physical activity, which is conducted within non-selected participants.

The primary aim of the present paper is to describe the effectiveness of a school-based physical activity intervention in enhancing executive functions and academic performance in adolescents. Furthermore, the effect of the intervention on physical activity levels, cardiorespiratory fitness and adiposity were assessed as these are potential explanatory variables.

## Methods

### Study design, setting and participants

The Learning, Cognition & Motion (LCoMotion) study was conducted as a cluster-randomized controlled trial with schools as the cluster unit of randomization. Inclusion of schools, rationale, aims and intervention elements is described in a previous publication [18]. The study included 16 schools of which 13 were recruited through an external collaborator [19]. This external collaborator was organizing a primary school project on physical activity and health at these schools. Classes included in this study were not involved in the primary school project. Further, three schools were included by volunteering. In order to enter the study, schools had to sign up classes from the 6^th^ and/or the 7^th^ grade (12–14 years old). No restrictions on the number of classes or students were given, but the majority of schools participated with all their grade 6 and/or grade 7 classes. Students were considered eligible if they followed normal age appropriate curriculum. The 16 schools were randomized to participate as either intervention (n = 9) or control (n = 7) schools. Included schools covered all five main regions of Denmark. The randomization process was conducted by the principal investigator as draws of folded paper with school names from a bowl in the presence of other senior researchers. All classes and students in a school were assigned to the group given by the school randomization. Two intervention schools withdrew the acceptance before the start of the study, but after the randomization. A total of 855 students from the remaining 14 school were considered eligible for inclusion in the study. The intervention took place from January to May 2014 (20 weeks). Baseline measures were performed at schools during November/December 2013 and follow-up measures during May/June 2014. Testing was balanced between control and intervention schools at both time points and all students at one school were tested during one day. Baseline measures were carried out after the randomization with schools aware of the randomization results. This was also known to the staff conducting the tests. Students participated in the study if they gave assent and one parent or legal guardian provided a written informed consent. Unwillingness to participate in the study did not influence students participating in class activities. The study was approved by the ethics committee of the region of Southern Denmark (S-20130104) and registered at clinicaltrials.gov (NCT02012881).

### Intervention components

The main aim of the intervention was to increase physical activity levels overall as well as in school by targeting classroom, recess and leisure-time activity and through active transportation. Specifically, schools made an oral agreement with the researchers to endeavor to provide 60 minutes of physical activity during school time on all school days during the intervention. A minimum of one teacher from each class at the intervention schools attended a course on incorporating physical activity into academic lessons. Courses were provided by an external collaborator (also responsible for inviting schools) and focused on practical didactic methods not specific to any given academic subject. The content of the course was delivered through theoretical and practical exercises by experienced educators with hands-on experience in integrating physical activity in academic lessons. The course did not target specific intensity levels of class-room physical activity, but was based on incorporating whole-body movement games into the academic curriculum. Scheduled activities during recess were initiated by teachers and volunteer students who received the course. Physical activity homework consisted of a booklet containing suggestions for various daily activities of 5–10 minutes, and students were instructed to perform at least one activity on all days. It was emphasized that these activities were in addition to usual daily activities. A two-week cycling campaign was launched in the middle of the intervention to facilitate active transportation by cycling. Finally, a custom made “activity watch” was used as a shared tool for teachers and students to serve as motivation and to sum up the amount of time the class had engaged in physical activity during academic subjects and scheduled recess activities. An overview of intervention components are given in Table 1. Control schools were asked to continue with their normal practice.

**Table 1:** Intervention content and means of implementation.

Intervention component	Practical organization	Responsible for conducting component	Implementation facilitated by and how	Period of implementation
Physical activity in academic subjects	Daily	Teachers	External collaborator: 3 hour course for teachers	Full intervention
Scheduled physical activity during recess	Weekly	Volunteer students & teachers	External collaborator: 4 hour course for students and teachers	Full intervention
Physical activity homework	Daily	Students	Research team: Monthly booklets	Full intervention
Active transportation	Daily	Students	Research team: Cycling campaign	2 weeks (intervention week 11 & 12)

### Measurements

The procedure was identical at all schools at both time points. During the first part of the day, students completed outcome measures (flanker task and mathematics performance, see below for description). The day was concluded with assessment of anthropometric variables and cardiorespiratory fitness.

### Cognitive control

A modified Eriksen flanker task was used to assess cognitive control [20, 21], which used five arrows to modulate interference control. In this task, a central, target arrow pointing to the right “>” required a right-handed response and a central, target arrow pointing to the left “<” required a left-handed response. These two response-eliciting stimuli were flanked by an array of other arrows that were congruent (>>>>> or <<<<<), or incongruent (>><>> or <<><<) to the centrally placed target stimulus. The incongruent condition has been shown to cause a delay in response time as well as a decrease in accuracy relative to the congruent condition, mainly because it requires greater amounts of inhibitory control to inhibit the interfering flanker stimuli and their associated response mappings. Each stimulus was presented for 120 milliseconds (ms). Students performed the task individually in small rooms provided by the school with a maximum of four students tested at the same time. Efforts were made to secure a similar testing environment across schools. Students performed the task under the guidance of a trained staff member who gave verbal instructions prior to testing. Testing conditions were identical at baseline and follow-up. The task consisted of 2 x 75 trials with congruent and incongruent trials being presented randomly and with equal probability. Response accuracy and reaction time (ms) data were collected as main outcomes of the trial. Response accuracy was defined as the percentage of correct responses while reaction time was defined as time to responds on correct trials. Both measures were summarized separately for congruent and incongruent trials. Only responses between 200–1470 ms post onset of stimuli were accepted. Further, differences (subtractions) in response accuracy and reaction time between congruent and incongruent conditions are presented as interference-scores [20, 22]. Students with an accuracy on either congruent or incongruent conditions <50% (less than change) were deemed to have misunderstood the task and their result were discarded from further analysis. Test-retest reliability of the flanker test was measured and is presented in S1 Table.

### Academic performance (mathematics skills)

Custom made grade specific mathematics tests were used to assess academic performance. The test was created by a content expert and included content such as; arithmetic’s, algebra, problem-solving and geometry. They were based on the national curriculum for the respective school year. The tests were administered in classrooms over 45 minutes by trained staff who gave identical instructions at all schools at baseline and follow-up. The scientific staff subsequently scored the tests. The tests consisted of 50 questions with possible scores ranging from 0 (worst) to 50 (best) points. No aids were permitted. Identical questions, but with numbers changed so answers were not the same, were administered post intervention. The tests were validated against an age-matched curricular test that is frequently used in the Danish public school system. During the pilot phase of the trial, the curricular test demonstrated a potential problem with floor-effects and tended to place students into two separate groups (bi-modal distribution) and was therefore not used. The custom made and the standard curricular tests were highly correlated (Spearman´s rank correlation coefficient 0.87, p<0.01) (18). Test-retest reliability of the custom made mathematics test is presented in S1 Table.

### Anthropometric and demographic variables

Body height was measured with a stadiometer (West Sussex, UK) to the nearest 0.5 cm. Body mass was measured to one decimal using an electronic scale (Tanita BWB-800, Tokyo, Japan). Overweight and obesity was defined according to age- and gender-specific BMI reference values [23]. Waist-circumference was measured to the nearest 0.5 cm just above the level of the umbilical cord with a minimum of two measurements performed. If these differed by more than 2 cm a third measurement was taken. The mean value of the two closest measurements was used as waist circumference. Students self-reported their pubertal development in a small questionnaire. This consisted of five categories of secondary sex characteristics as defined by Tanner [24]. Baseline scores of pubic hair for boys and breast development for girls were used. Ethnic origin and socioeconomic status was obtained from a questionnaire answered by a parent or legal guardian. Maternal or female guardian’s highest completed education was used as indicator of socioeconomic status.

### Cardiorespiratory fitness

Cardiorespiratory fitness was assessed by the Andersen test [25]. This is a 10-min intermittent running test that can be easily conducted at schools. The test has been validated against direct measures of maximum oxygen uptake in different age groups and found to give valid and reliable results [25, 26]. The total distance in meters was used as test result. Cardiorespiratory fitness was not included from one control school as it was not possible to conduct the test in accordance with the protocol. Changes in fitness of more than two standard deviations for gender and intervention group were inspected as this might indicate an invalid test at either time point. In case of a clear submaximal performance at one time point, the results were discarded (24 observations).

### Physical activity levels

Physical activity levels of participants were assessed by accelerometry before and during the third month of the intervention. GT3X and GT3X+ devices (ActiGraph LLC, Pensacola, FL, USA) were distributed by research staff to students at schools with the instruction to wear the devices on the right hip every day during a seven day period. Due to a shortage of devices, one intervention school did not receive accelerometers, as this school entered the study just prior to baseline testing. Furthermore, accelerometers were only distributed in four of five, four of six, and three of four classes at another three schools (all control schools), respectively. The epoch was set to two seconds but files were downloaded in ten seconds epochs. The software program Propero (University of Southern Denmark, Odense, Denmark) was used to summarize counts and time recorded from 06:00–22:00 hours. A sequence of more than 30 minutes of consecutive zeroes were considered non-wear time and was not included in analyses. To be included, students had to obtain a minimum of four days with at least ten hours of valid registration at both time points. The intensity threshold suggested by Evensson [27] was used to summarize daily minutes spent in moderate to vigorous physical activity (MVPA). The cut-point was divided by 1.5 to accommodate the difference in epoch length between the Evensson study and this study (15 vs. 10 sec.) [28, 29]. As a measure of average physical activity level, total counts were divided by wear time to generate mean counts/min (cpm). Class-specific time-tables were used to generate the domains; total school time, class-time (excluding physical education) and recess. Estimates of mean count and MVPA were derived using the same procedure except that intensity domains were expressed as percentage of recorded time to accommodate differences in bell ringing hours. All summaries were completed separately for each day and aggregated to give the final result.

### Short message service-tracking

Every second week of the intervention period information on compliance with the intervention elements was collected using short message service (SMS)-tracking system on mobile phones (automatic text messaging) during every second week of the intervention period. Students at intervention schools received a message every second Friday asking how many times in the last week:

- They had participated in scheduled recess activities
- Their teachers had initiated physical activities during academic subjects
- They had conducted physical activity homework

Every 28 days students at both intervention and control schools were asked about bicycling to and from school during the present week.

Main classroom teachers in all intervention classes were sent one question each week:

- How many minutes had the class been physically active in the past week according to the “activity watch”?

### Statistics

An a priori calculation of sample size was carried out. In order to detect an intervention effect (two-sided significance test) of three percentage points on accuracy of incongruent flanker trials with a power of 80%, alpha at 5%, an assumed cluster-effect of 0.01 and an average of 80 students per school, a total of 680 students were required. Analyses of intervention effects were performed with regard to intervention arm (intervention or control) irrespective of compliance. Only students obtaining acceptable flanker task performance or mathematics skills assessments at both time points were included (complete-case analysis). Differences in change (follow-up–baseline) were used as outcome measure and reported as beta coefficients (β) from linear regression models adjusted for baseline values of the relevant outcome, for gender and for grade. As the units of randomization were schools a “random effect” was added by using mixed models (maximum likelihood based) to accommodate the clustering of students within these units. If a random intercept for school and/or class did not improve the model fit according to the likelihood ratio test, they were removed and an ordinary least squares regression was used. To facilitate comparison across studies, standardized coefficients were additionally calculated for cognitive and academic outcomes by standardizing the relevant outcome to its mean and standard deviation prior to running the linear regression model. Standardized coefficients are expressed so positive values represent changes favoring the intervention group. Potential differential intervention effects between boys and girls were analyzed by including an interaction term between gender and type of school (intervention or control). A significance level of 0.1 was used to evaluate the interaction term. In case of significance, analyses were stratified by gender and rerun. Differences in change in waist-circumference, BMI, cardiorespiratory fitness and physical activity were investigated in a similar manner. Potential differences in baseline values between drop-outs and the final sample were investigated using linear regression with adjustment for gender and grade for continues variables and with Chi^2^ tests for categorical variables. Baseline differences between students at intervention and control schools were investigated using linear or logistic regression (maximum likelihood based) adjusted for gender and grade if appropriate. Chi^2^ was used for categorical variables not influenced by gender and age. The same was done to investigate differences between students with and without assessment of objectively measured physical activity. Similarly to the analysis of change, random effects terms for school or class were used in drop-out analyses and baseline comparison if they improved the fit of linear or logistic models.

Despite the randomized design, differences in the distribution of ethnic origin, grade, status of overweight at baseline in boys, and pubertal development (when adjusted for age) differed at baseline between the groups. It was therefore investigated whether these variables were possible confounders by assessing their association with outcomes (changes) irrespective of group. Based on these analyses, only overweight status was included in the cardiorespiratory fitness model while grade was included in all models. Finally, analyses were rerun with and without the possible confounders and results were not noticeably different. As a sensitivity analysis to account for loss to follow-up between baseline and follow-up, multiple imputations were performed on all missing outcome variables. These data are presented in S2 Table. Imputation of variables was performed by using chained equations (“mi impute chained”) in Stata v13.1. Beta coefficients and standard errors were obtained based on 20 imputed datasets. Imputation models were visually checked for convergence and the reproducibility of the estimates were inspected by Monte Carlo errors. The imputation analyses are based on the assumption of data being missing at random conditional on the observed covariates (missing at random). Conclusions from the imputation analyses did not differ from analyses of complete data. Linear models were investigated for assumptions of residual normality in overall model, for normality of the random intercepts if appropriate and for homoscedacity of the residuals across the groups. Appropriate assumptions for other models were assessed as well. No evidence of failure to comply with assumptions was found in any of the statistical models. All analyses were conducted using Stata IC v.13.1 (StataCorp, College Station, Texas, USA) with α = 0.05 (two-sided). A CONSORT checklist is available in S3 Table.

## Results

**Fig 1:**
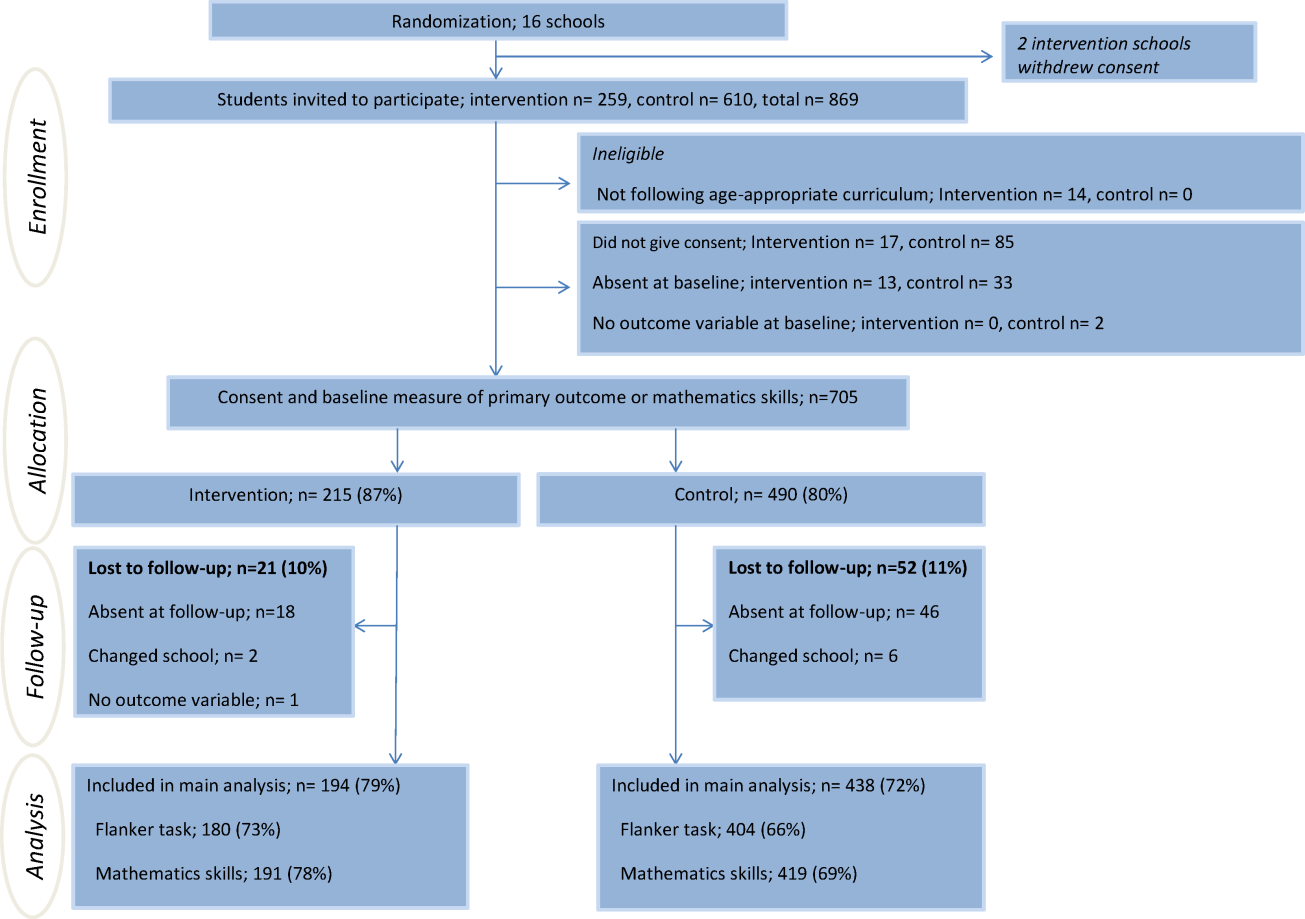
Participant flowchart. Numbers in percentage are of eligible students except for percentage lost to follow-up, which is of consenting students with a baseline measure of the primary outcome or mathematics skills. Consenting students differ from numbers published previously [18] as student’s not following age appropriate curriculum (n = 14) were not included in the trial. The flow of schools is presented in [18].

Forty classes at the 14 schools participated as intervention or control schools with 12 participating as intervention and 28 as controls. The median (25–75 IQR) number of participating students from these classes was 16 (14–19) and 17 (15–19), respectively. In total, 632 consenting students (194 at intervention and 438 at control schools), had minimum one outcome measurement at both time points and comprised the final sample (74% of randomized subjects). Of these, 584 students had an acceptable flanker task performance at both time points and 622 completed the mathematics skills test at both time points (for participant flow after randomization see Fig 1). The main reason for missing data on the flanker task was accuracy below chance (35 at baseline and 4 at follow-up). Data was missing on the mathematics test for 14 students at baseline and 8 students at follow-up).

### Loss to follow-up

Significant differences at baseline existed between students included in analyses and those lost to follow-up (baseline, but no follow-up outcome measurement, n = 73; 10% of baseline sample). The included sample was younger, had higher cardiorespiratory fitness, lower waist-circumference, lower BMI, and was more likely to be of “other ethnic origin” (all p’s≤0.05). No significant differences were found between the included sample and those lost to follow-up in the flanker task, mathematics performance, the distribution of gender, pubertal development or socioeconomic status (p’s>0.05). Among students lost to follow-up, significant differences between intervention and control school students (n = 21 and n = 52, respectively) were found for cardiorespiratory fitness (869 vs. 1012 meters, p<0.01), waist-circumference (71 vs. 80 cm, p<0.01) and BMI (19.8 vs. 23.1, p = 0.01). All values favored control schools. Age was higher in control school children (13.2 vs. 12.9 years, p = 0.05) who were lost to follow-up compared to intervention school children lost to follow-up.

### Baseline characteristics

Baseline characteristics are depicted in Table 2. At baseline, students at intervention schools were younger (p = 0.05), less fit (difference = 33 meters, p = 0.02) and had a higher BMI (p = 0.05) compared to students at control schools. There were no differences in the overall proportion of overweight or obese students (2% were obese), but a significantly higher proportion of boys at intervention schools were overweight (p = 0.05) when compared to boys from the control schools. Furthermore, students at intervention schools reported being significantly further along in pubertal development (p = 0.05) and a significantly higher proportion of students at intervention schools were of ethnic Danish origin (p = 0.02). No significant baseline differences were observed in the flanker task, mathematics skills or waist-circumference (all p’s>0.05).

**Table 2:** Baseline characteristics of participants by group allocation and gender. Values are numbers (percentages) unless stated otherwise.

	Intervention schools		Control schools		p-values for difference between intervention and control groups at baseline
	Boys n = 100	Girls n = 94	Boys n = 209	Girls n = 229	0.37
**Mean (SD) age (years)**^**a**^	12.8 (0.6)	12.7 (0.5)	13.1 (0.6)	12.9 (0.6)	<0.001
**Overweight or obese**^**b**^^,^ ^**c**^	19 (19)	14 (15)	24 (12)	32 (15)	0.28
**6th grade students**^**d**^	82 (82)	79 (84)	106 (51)	134 (59)	<0.001
**Pubertal development**^**e**^^,^ ^**f**^					0.045
I + II	30 (32)	3 (3)	58 (29)	15 (7)	
III	32 (34)	50 (56)	84 (42)	115 (53)	
IV + V	32 (34)	36 (40)	60 (30)	88 (40)	
**Socioeconomic status**^**d**^^,^ ^**g**^					0.35
I	11 (12)	10 (12)	35 (19)	28 (14)	
II	48 (53)	35 (41)	71 (39)	95 (47)	
III	31 (34)	41 (48)	75 (41)	77 (39)	
**Ethnic origin**^**d**^					0.02
Danish	88 (98)	82 (96)	172 (94)	183 (91)	
European	2 (2)	2 (2)	3 (2)	5 (2)	
Other	0 (0)	1 (1)	8 (4)	14 (7)	

Eighteen parents (9%) from intervention and 54 (12%) parents from control schools did not return the questionnaire and information on socioeconomic status and ethnic origin is missing for these students. Likewise, age, pubertal development and BMI at baseline was missing for 1 (1%), 11 (6%) and 2 (1) of intervention school students, respectively and 4 (1%), 18 (4%) and 11 (3%) of control school students.

^a^p-value is from a linear regression model regressing age on intervention group.

^b^Overweight and obesity was defined according to age- and gender specific BMI reference values [23].

^c^p-value is from a mixed logistic model with class as a random effect.

^d^p-value is from a Chi^2^ test.

^e^Secondary sex characteristics according to Tanner with I+II and IV+V combined because of few observations in category I and V. Category I means not started puberty, Category V is matured as an adult.

^f^p-value is from a mixed ordinal logistic model adjusted for age and gender with class as a random effect.

^g^Maternal or female guardians highest completed education was used as the socioeconomic status indicator. I is no formal education, II is vocational training or < 3.5 years of adult education and III is≥ 3.5 years of adult education (bachelor-level).

### Intervention effects–cognitive and academic performance

The flanker task was successful in decreasing accuracy and increasing reaction time on incongruent relative to congruent trials at baseline and at follow-up. No significant differences in change between intervention and control schools were observed for response accuracy or reaction time on congruent or incongruent trials (results are presented in Table 3). Standardized coefficients (95% confidence intervals (CI)) were -0.06 (-0.19–0.07) and 0.08 (-0.07–0.23) for congruent and incongruent response accuracy and -0.05 (-0.21–0.11) and -0.07 (-0.23–0.09) for congruent and incongruent reaction time. Likewise, no significant difference was observed for accuracy interference scores. However, a significant effect in favor of control schools was found on reaction time interference scores (adjusted β = 5.0, p = 0.03) such that control schools experienced a larger reduction in interference score compared to intervention schools. The standardized coefficients (95% CI) were 0.12 (-0.03–0.26) and -0.15 (-0.29–0.01) for accuracy and reaction time interference scores, respectively. No significant effect of the intervention was observed on mathematics skills. The standardized coefficient (95% CI) for mathematics skills was -0.05 (-0.31–0.21).

**Table 3:** Intervention effects on the flanker task and academic performance.

	Intervention				Control							
Outcome	n =	Baseline	Follow-up	Within-group change	n =	Baseline	Follow-up	Within-group change	Adjusted difference in change^a^	95% CI	p-value	ConditionalICC
Flanker task												
**Accuracy (%)**												
Congruent trials	180	94.8 (6.1)	96.7 (5.2)	1.8 (5.2)	404	94.9 (6.2)	96.9 (4.3)	2.0 (5.5)	-0.3	-1.0–0.4	0.36	NA
incongruent trials	180	80.9 (11.9)	86.6 (10.1)	5.7 (9.3)	404	83.2 (11.9)	87.0 (10.4)	3.8 (10.3)	0.8	-0.7–2.3	0.31	NA
Interference score	180	14.0 (9.5)	10.1 (7.6)	-3.9 (7.9)	404	11.8 (9.2)	10.0 (8.6)	-1.8 (9.1)	-1.0	-2.3–0.3	0.13	NA
**Reaction time (ms)**												
Congruent trials	180	470.2 (74.6)	458.9 (61.7)	-11.3 (42.7)	404	461.9 (64.1)	449.8 (61.2)	-12.1 (50.2)	2	-5–10	0.57	NA
incongruent trials	180	557.4 (92.4)	534.4 (70.8)	-23.0 (54.9)	404	541.2 (81.5)	516.6 (70.3)	-24.7 (59.1)	6	-2–15	0.16	NA
Interference score	180	87.2 (41.7)	75.4 (31.1)	-11.7 (34.0)	404	79.3 (35.3)	66.7 (28.3)	-12.6 (32.5)	5	0–9	0.03	NA
Academic performance												
Mathematics skills^b^	191	19.5 (10.2)	22.1 (10.9)	2.6 (5.4)	419	20.3 (9.9)	22.8 (11.1)	2.4 (4.9)	-0.2	-1.6–1.1	0.73	0.08 (class)

Baseline, follow-up and within-group changes are unadjusted values while between-group comparisons are adjusted. Values are mean (SD) unless stated otherwise. Mathematics performance can range from 0–50 points. RT: reaction time, CI: confidence interval, ICC: Intraclass correlation coefficient conditional on covariables. NA: not applicable. NOTE: A negative difference in change for accuracy means worse performance by intervention schools (smaller increase in accuracy from baseline to post-intervention). A positive difference in change for RT means worse performance by interventions schools (smaller decrease in response speed from baseline to post-intervention). A positive difference in change in interference scores means worse performance by intervention schools (smaller decrease in interference score).

^a^ Estimates are the unstandardized beta-coefficients (control coded as zero, intervention coded as 1) from linear mixed models with the change in outcome adjusted for baseline value of outcome, gender and grade level. If a random effect did not improve the model fit, ordinary least squares regression was used.

^b^ Between-group difference in change is further adjusted for class cluster as a random effect.

A significant interaction was observed between gender and school type for mathematics performance (p = 0.01 for interaction). However, in gender stratified analysis there were no significant differences between intervention or control school students in either gender (boys: adjusted β = -1.10, p = 0.18 & girls: adjusted β = 0.57, p = 0.52). The standardized coefficients (95% CI) for boys and girls were -0.20 (-0.49–0.09) and 0.12 (-0.24–0.48), respectively.

### Intervention effects–anthropometrics and cardiorespiratory fitness

No significant differences in change between intervention and control schools were found for BMI, waist-circumference or cardiorespiratory fitness (presented in Table 4). Interactions were observed between gender and school type for BMI (p = 0.03 for interaction), waist-circumference (p<0.01 for interaction) and cardiorespiratory fitness (p = 0.03 for interaction). In stratified analyses boys at intervention schools had a significantly smaller increase in BMI compared to boys at control schools (adjusted β = -0.22, p = 0.01) while there were no significant differences with respect to waist circumference (adjusted β = -0.49, p = 0.11) or cardiorespiratory fitness (adjusted β = -3.7, p = 0.71). Girls at intervention schools had a significantly larger increase in cardiorespiratory fitness compared to girls at control schools (adjusted β = 21.5 p = 0.01) while there were no significant differences with respect to BMI (adjusted β = 0.0, p = 0.72) or waist-circumference (adjusted β = 1.35, p = 0.38).

**Table 4:** Intervention effects on cardiorespiratory fitness and anthropometric variables.

	Intervention				Control							
Outcome	n =	Baseline	Follow-up	Within-group change	n =	Baseline	Follow-up	Within-group change	Adjusted difference in change^a^	95% CI	p-value	Conditional ICC
**Cardiorespiratory fitness (distance, m)**^**b**^	162	989 (107)	1018 (109)	29 (57)	297	1023 (114)	1040 (19)	17 (72)	9.4	-3.7–22.4	0.16	NA
**Waist circumference (cm)**^**c**^	190	71.6 (8.4)	72.8 (8.4)	1.2 (4.1)	410	70.4 (8.9)	70.5 (8.7)	0.2 (2.9)	0.7	-0.7–2.1	0.33	0.13 (school)
**BMI**	191	19.8 (2.9)	20.1 (2.9)	0.3 (0.7)	417	19.3 (3.0)	19.7 (3.1)	0.4 (0.7)	-0.1	-0.2–0.0	0.14	NA

Baseline, follow-up and within-group changes are unadjusted values while between-group comparisons are adjusted. Values are mean (SD) unless stated otherwise. CI: confidence interval. BMI: body mass index. NA: not applicable. On the basis of changes of >2 standard deviations, 24 participants were further excluded from the cardiorespiratory fitness results (five at intervention and 19 at control schools).

^a^ Estimates are the unstandardized beta-coefficients (control coded as zero, intervention coded as 1) from linear mixed models with the change in outcome adjusted for baseline value of outcome, gender and grade level. If a random effect did not improve the model fit, ordinary least squares regression was used.

^b^ Between-group difference in change is further adjusted for overweight status at baseline.

^c^ Between-group difference in change further adjusted for school cluster as a random effect.

### Physical activity levels

In total, 638 participants were given the opportunity to wear an accelerometer. Of these 245 students (38%) had a valid physical activity measurement at both time points. Upon inspection of model assumptions one extreme observation (standardized residual = 5.17) was dropped. Therefore 244 students comprised the final physical activity sample. Students with physical activity data differed from students without physical activity data with respect to mathematics performance, grade, sex and ethnicity (all p’s≤0.05).

### Intervention effects on physical activity

No significant differences between intervention and control schools were found for total wear time (p’s>0.17) or the number of weekend days included (p’s>0.84) at either time point. Likewise, no significant differences in activity levels were found at baseline (p’s>0.16).

Changes from baseline to mid-intervention in overall physical activity levels or daily minutes spent at MVPA did not differ significantly between intervention and control schools. Nor did they differ in any of the specified subdomains (presented in Table 5). No interaction between gender and school type was evident in any models.

**Table 5:** Intervention effects on physical activity according to group allocation.

	Intervention (N = 96)			Control (N = 148)						
Outcome	Baseline	Mid-intervention	Within-group change	Baseline	Mid-intervention	Within-group change	Adjusted difference in change^a^	95% CI	p-value	ConditionalICC
**Physical activity level (cpm)**^**b**^	394 372–417	462 435–491	73 (111)	386 364–409	428 403–455	47 (133)	5	-30–41	0.77	Class: 0.04
**Overall MVPA (minutes/day)**^**b**^	44.9 41.9–48.3	55.2 51.2–59.6	11.4 (17.2)	44.5 41.6–47.7	51.5 48.2–55.1	7.4 (17.4)	1.2	-3.9–6.3	0.64	Class: 0.04
**All school time (cpm)**^**b**^	465 436–496	492 461–525	29 (152)	405 382–429	422 393–453	30 (156)	1	-62–63	0.98	Class: 0.24
**All school time MVPA (%)**^**b**^	6.7 6.2–7.3	7.3 6.7–7.9	0.7 (2.8)	5.9 5.5–6.3	6.4 5.8–6.9	0.9 (2.9)	-0.3	-1.5–0.9	0.63	Class: 0.25
**Class time (cpm)**^**b**^	291 271–312	288 268–309	-2 (108)	25 9 243–277	250 231–271	0.9 (131)	6	-53–64	0.86	Class: 0.42
**Class time MVPA (%)**^**b**^	3.6 3.3–4.0	3.7 3.3–4.0	0.0 (2.0)	3.2 2.9–3.5	3.1 2.8–3.5	0.2 (2.3)	0.0	-1.0–1.0	0.98	Class: 0.37
**Recess (cpm)**^**b**^^,^ ^**c**^	903 822–992	982 893–1080	89 (462)	691 631–757	690 619–769	52 (458)	112	-84–308	0.26	School: 0.12. Class 0.04
**Recess MVPA (%)**^**b**^^,^ ^**c**^	14.3 12.8–15.9	15.7 13.9–17.7	1.8 (8.2)	10.7 9.6–11.9	10.6 9.2–12.3	1.6 (9.4)	1.2	-3.2–5.6	0.59	School: 0.17. Class: 0.07

Baseline, follow-up and within-group changes are unadjusted values while between-group comparisons are adjusted. All physical activity values were skewed hence baseline and mid-intervention values are presented as geometric means with 95% CI ratios. Changes are presented as means with SD for within group-changes and means with 95% CI for between group changes.

^a^ Estimates are the unstandardized beta-coefficients (control coded as zero, intervention coded as 1) from linear mixed models with the change in outcome adjusted for baseline value of outcome, gender and grade level. If a random effect did not improve the model fit, ordinary least squares regression was used.

^b^ Between-group difference in change is further adjusted for class cluster as a random effect.

^c^ Between-group difference in change is further adjusted for school cluster as a random effect.

CI: confidence interval, MVPA = moderate to vigorous physical activity, CI = confidence interval, ICC: Intraclass correlation coefficient.

### SMS-tracking

**Fig 2:**
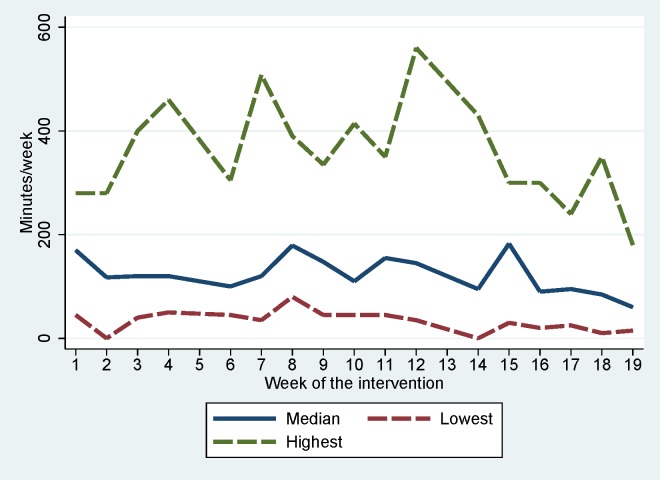
Self-reported class-room and scheduled recess physical activity in the intervention group. Minutes of physical activity/week reported on the “activity watch” by teachers in intervention classes during the intervention period. For each week the median, the lowest and the highest class values are depicted. Interventions week 5 and 13 are holidays but lines are drawn through for graphical appearances.

The median (25–75 IQR) weekly number of “activity watch” responses was 11 (10–12) with a median (25–75 IQR) weekly physical activity reporting of 120 minutes (73–240). This corresponds to 24 minutes of classroom based or scheduled recess activities per day during the intervention period. The use of the “activity watch” during the intervention is depicted in Fig 2.

In total, 177 (91%) students from intervention schools agreed to participate in the SMS-tracking system. Responses varied at each SMS round from 79% to 93%. Implementation and progression of individual intervention components is presented in S1 File.

## Discussion

The data did not support an effect of a 20-week multi-faceted physical activity intervention on the a priori specified primary outcomes on a task of executive control, however the intervention was limited by poor fidelity which makes a causal interpretation of the effects of regular physical activity on cognitive and academic outcomes tenuous. Accordingly, this study provides three important lessons. Firstly, careful and detailed information about the actual physical activity performed is paramount in settings where tight control is difficult, as there is little reason to expect the outcome to change if there is no change in exposure level. Multiple physical activity interventions have not been able to demonstrate increased overall levels of physical activity [30–32]. Secondly, in the present study, the objective information on physical activity from accelerometers supports a different conclusion than the subjective data from SMS-tracking. This underpins the need for objective assessments as these are generally considered less prone to differential error or social desirability bias in comparison with subjective measures [33]. Thirdly, future trials should consider including a qualitative assessment of intervention fidelity, possibly in combination with a pilot-phase to develop and improve intervention feasibility and fidelity. This is however not a guarantee for a successful trial [32]. The combination of objective physical activity assessment and qualitative data demonstrating intervention fidelity would provide the optimal opportunity to evaluate an intervention. Further, qualitative data on factors related to intervention fidelity can be highly informative when improving existing programs or implementing a successful program.

As was the case in the present study, modified versions of the Eriksen flanker task are widely used as measures of inhibitory control [15, 21], which is one of three core constructs of executive functions [8]. The flanker task has previously been demonstrated to be responsive to changes in physical activity exposure in randomized controlled trials using both longitudinal [15] and acute study designs [34]. The lack of an intervention effect on the primary outcomes and the greater change in interference control in the control group compared to the intervention group is discordant with existing evidence from previous randomized controlled trials. Two afterschool physical activity programs have demonstrated positive benefits on cognitive outcomes within a time-span of three to nine months [14, 15]. Similarly, small school-based trials with manipulation of physical activity during physical education classes have demonstrated favorable intervention effects within four months in both children [35] and adolescents [36]. In the light of previous literature and the multiple tests performed, it is possible that the finding on interference scores is spurious. When a post hoc re-analysis of the data was performed with 111 intervention and control group participants that could be matched 1:1 for gender, age, pubertal development, ethnicity, cardiorespiratory fitness, waist-circumference and mathematics score, this gave identical results to the a priori specified analysis, except that changes in interference control did no longer statistically favor the control group (data not shown). Further, the observed difference in change was 5 ms but the clinical importance of such a difference is unknown. Important conceptual differences exist between the afterschool interventions and the present trial. These include the highly controlled form (led by physical activity specialists) and the inclusion of pre-pubertal children whereas this trial was delivered by non-specialized teachers within the school day and included adolescents, who were at a later stage of puberty. These differences make a direct comparison difficult while previous school-based trials are limited by their sample sizes. Established correlates of greater cognitive function such as high cardiorespiratory fitness [5], low body fat [7] and a favorable cardiometabolic risk score [22] are all favoring a physically active lifestyle. However, the association between objectively measured physical activity and cognitive performance is unclear with previous research demonstrating both a positive association [37] and no association [38].

In this study girls at intervention schools improved their cardiorespiratory fitness relative to girls at control schools, while boys at intervention schools had a relative improvement (smaller gain) in BMI compared to boys at control schools. Given the abovementioned correlates, an effect on the primary outcome might have been expected. The effect of the intervention on cardiorespiratory fitness of girls was 21.5 meters, which corresponds to 2% of the average distance covered by girls at baseline. A significant effect on inhibitory control was found in conjunction with a 4% greater improvement in directly measured maximal oxygen uptake in the intervention group compared to the control group in the FITKids trial [15]. Similarly, the intervention effect on BMI in boys (0.22 kg/m^2^) was also smaller than in the FITKids trials (0.5 kg/m^2^). A possible explanation is that the current effects on adiposity and cardiorespiratory fitness were insufficient for a positive effect on inhibitory control to be observed. Effects on cardiorespiratory fitness or adiposity were not assessed in the trial by Davis and colleagues [14]. Further, an important distinction from the other trials is that only ≈ 15% of the study sample were overweight. The potential for change may thus have been different. A final note on the significant intervention effects is that the effects were observed on change scores but that the post-intervention BMI of intervention school boys or cardiorespiratory fitness of intervention school girls where not different from the BMI of boys at control schools or the cardiorespiratory fitness of girls at control schools.

Only a few intervention studies with an academic performance outcome exist and even fewer are school-based with randomized designs [4]. Three school-based interventions are roughly comparable in design (randomized intervention with pre-post measures) but differ in duration, outcome(s) and included age-group [16, 39, 40]. Results from these studies are inconsistent regarding effects on academic achievement, with only the PAAC study [16] demonstrating a positive effect. In that study, a significant difference in three-year changes in several areas of academic achievement was found. However, academic achievement was a secondary outcome and was evaluated in a subsample only. The study by Ahamed and colleagues [40] did not find a significant difference in performance on an aggregated test of academic achievement, while Sallis and colleagues [39] found mixed results in different areas of academic achievement. Therefore, available evidence suggests that school-based physical activity interventions, despite not enhancing academic achievement, have no negative influence on academic achievement, indicating that time taken away from academics in favor of physical activity do not come at the cost of academic outcomes.

### Implementation of the intervention

The “activity watch” revealed that time directed towards physical activity was integrated into academic subjects. However, as no information on baseline or control school levels of academic subject activity levels is available, this does not permit the conclusion that intervention schools had a higher physical activity level during academic subjects than control schools. In fact, this was not supported by data from accelerometers. Furthermore, the data did not suggest that the activity levels in academic subjects had been increased at intervention schools compared to pre-intervention. This is surprising as this was a key element of the intervention and it should be kept in mind that accelerometers only assess a given period of time whereas the SMS-track surveyed the entire intervention period. Comparing results from the “activity watch” during the weeks of accelerometer wear to the rest of the intervention period did not however indicate the surveyed week differed substantially from the “usual” week (135 minutes vs. 120 minutes during the entire intervention). In summary, the data do not support that the aim of delivering 60 minutes of physical activity on each school day was reached. The “activity watch” demonstrates that the implementation of the intervention was quite stable over time with indication of a leveling off of the intervention during the last few weeks. It is also clear that compliance with the intervention was not universal but differed greatly between classes. Results from students text messaging demonstrate a similar picture (S1 File). Two other school-based physical activity interventions also included physical activity during academic subjects as a core intervention component. In the PAAC study teachers delivered ≈ 75 minutes of physical activity during academic subjects per week, while Ahamed et al (2007) reported that intervention schools spent on average 50 more minutes of physical activity in class per week. Both used self-report methods. Our result of 120 minutes/week is somewhat higher but also includes structured recess activities. Importantly, none of the other studies performed an objective measure of in-class physical activity levels.

### Strengths and limitations

Strengths of the study include the relatively large sample size, the use of a cluster randomized design and the inclusion of objective measures of physical activity. The main limitation of the study is the poor intervention fidelity. An explanation could be that 20 weeks was an insufficient amount of time to implement and evaluate the effects of a physical activity intervention within academic subjects. Furthermore, schools were recruited through a primary school physical activity and health project, which could be considered a limitation, as the included schools may have a physical activity awareness, which differs from other public schools in Denmark. Possibly, the degree of changes to implement at intervention schools may have been reduced. Also, the differences observed at baseline indicate that the randomization process did not result in completely comparable groups. As the number of clusters was limited (14 schools) some differences after randomization are not unexpected. Potential confounders (unevenly distributed between groups) were omitted if unimportant (not related to outcome) and otherwise adjusted for statistically. Potential confounders included pubertal development, ethnicity and weight status, which all had negligible effects on changes in outcome measures. Only one measure of cognitive and academic performance was included, which only provides a small snapshot into the potential relation that physical activity may have on the developing brain. Future studies should seek to include a broader view by including multiple measures of cognition and academic performance. Further, the use of standardized measures of academic performance should be included when available to facilitate comparisons between studies.

Two points in randomized design concern the internal validity of the study; 1) the loss to follow-up after randomization but before baseline-measure and 2) the loss to follow-up after baseline-measures, but before follow-up. The implication of this missing data at each point is the same, but no information is available on those lost at point 1 while baseline data is available for those lost at point 2. It was chosen to perform complete-case analysis as the primary analyses instead of using the intention-to-treat approach as this requires analyses of all those randomized to give an unbiased estimate of group allocation [41]. However, this would not be possible due to the loss to follow-up in point 1. This is a limitation of this study that was based on logistical reasons. The results of the imputation analyses (presented in S2 Table), which analyzed all students with a baseline measure did not lead to different conclusions, suggesting that loss to follow-up after baseline measures was not a source of bias which affected the study results.

Finally, as the adherence to the accelerometer protocol was low (38%) it may be subject to bias. This limits our ability to determine whether the non-significant finding on the primary outcome owes to a true lack of effect or whether the intervention did not create large enough differences between intervention and control schools to be of importance.

## Conclusions

No evidence was found for effectiveness of a 20-week physical activity intervention on increasing performance in an executive functions test or a mathematics skills test in Danish adolescents. However physical activity levels were not affected by the intervention, which makes it impossible to conclude on the causal relationship between increased physical activity and cognitive and academic performance. Positive results of the intervention include cardiorespiratory fitness in girls and BMI in boys, while a negative result was found for interference control. Improvements in cardiorespiratory fitness and BMI were only modest in comparison with improvements observed in successful trials. Such results speak to the importance of efficacious intervention techniques to improve not only physical health outcomes, but cognitive and scholastic health outcomes as well.

## Supporting Information

S1 File Implementation of intervention components. SMS-tracking from students at intervention schools.
(DOCX)

S2 File Trial protocol_englishVersion. Approved trial protocol (English translation).
(PDF)

S3 File Trial protocol_Danishversion. Approved trial protocol (Danish).
(PDF)

S4 File Bugge 2014—LCoMotion—Learning, Cognition and Motion; a multicomponent cluster randomized school-based intervention aimed at increasing learning and cognition—rationale, design and methods. Published trial protocol.
(PDF)

S1 Table Reliability measures. Test-retest reliability of the flanker task and mathematics skills.
(DOCX)

S2 Table Multiple imputation analyses. Results of multiple imputation analyses.
(DOCX)

S3 Table CONSORT Checklist. CONSORT 2010 checklist of information.
(DOC)

## Abbreviations

BMI: Body mass index
MS: Milliseconds
CI: Confidence interval
CPM: Counts per minute
ICC: Intraclass correlation coefficient
MVPA: Moderate to vigorous physical activity
RT: Reaction time
SD: Standard deviation
