# Supplementary material for: Effectiveness of a School-Based Physical Activity Intervention on Cognitive Performance in Danish Adolescents: LCoMotion—Learning, Cognition and Motion – A Cluster Randomized Controlled Trial
Source: PLoS One. 2016 Jun 24;11(6):e0158087. doi: 10.1371/journal.pone.0158087 (PMC4920412; doi:10.1371/journal.pone.0158087)
Supplement: S1 File — SMS-tracking from students at intervention schools. (DOCX) [file pone.0158087.s001.docx]

**S1 File. Implementation of intervention components.** SMS-tracking from students at intervention schools.

Activities at schools

Implementation and progression of the intervention components as reported by students on the SMS-track is presented in figures S4.1, S4.2 and S4.3. In total, 177 (91%) students from intervention schools agreed to be sent SMS-messages with responses varying at each SMS round from 140 – 165 (79% to 93%). At each round the median (low – high interval) percentage of students reporting to have performed PA homework > 4 times was 36% (30 % - 52%) while 24% (21% -40%) reported to have participated in physical activity during academic subjects >4 times. At each round 61% (55% - 71%) reported no engagement in the structured recess component.

Figure S4.1. Implementation of physical activity homework

Percentage of students in categories of compliance at biweekly SMS rounds during the intervention period. The numbers in the bars are percentage in the category. No restrictions on the number of answers to be included were used.

Figure S4.2. Implementation of physical activity during academic subjects

Percentage of students in categories of compliance at biweekly SMS rounds during the intervention period. The numbers in the bars are percentage in the category. No restrictions on the number of answers to be included were used.

Figure S4.3. Implementation of structured recess activities

Percentage of students in categories of compliance at biweekly SMS rounds during the intervention period. The numbers in the bars are percentage in the category. No restrictions on the number of answers to be included were used.

Use of cycling to school

Every 28 days of the study (Fridays at 5 pm) students were sent and SMS asking about their use of cycling to and from school during the present week. During week 11 and 12 of the intervention a cycling campaign was launched at intervention schools in order to promote cycling to and from school. The percentages of cycling student at intervention and control schools, as well as the results of the cycling campaign, are presented in table S4.1. A total of 564 students (177 intervention (91%) and 387 (88%) from control schools) answered an SMS-message about cycling to school at one time point. No restrictions on the number of answers to be included were used Questions on cycling during week 12 were only sent to students at intervention schools to continue motivation for the campaign. Correlation between school cycling to and from school was substantial (Spearman’s rho >0.94 at each week) so only cycling to school is used.

Table S4.1. Cycling to school

| Intervention week | Responses given (n) | Intervention (%) | Control  (%) | P-values for difference  (chi^2^) |
| --- | --- | --- | --- | --- |
| 2 | 451 | 17.09 | 34.13 | <0.00 |
| 6 | 512 | 29.59 | 51.31 | <0.00 |
| 11 | 503 | 54.49 | 55.06 | 0.91 |
| 12 | 149 | 51.68 |  |  |
| 15 | 476 | 44.44 | 61.30 | <0.01 |
| 18 | 493 | 42.11 | 61.00 | <0.00 |
|  | Total: 564 | 177 | 387 |  |

Percentage of students reporting to have cycled to school >2 times at intervention and control schools by week of the intervention. The campaign to promote school cycling was initiated in week 11 through 12.
