# Supplementary material for: Effectiveness of a School-Based Physical Activity Intervention on Cognitive Performance in Danish Adolescents: LCoMotion—Learning, Cognition and Motion – A Cluster Randomized Controlled Trial
Source: PLoS One. 2016 Jun 24;11(6):e0158087. doi: 10.1371/journal.pone.0158087 (PMC4920412; doi:10.1371/journal.pone.0158087)
Supplement: S2 File — Approved trial protocol (English translation). (PDF) [file pone.0158087.s002.pdf]

Ethics committee of Southern Denmark
Regionshuset, Damhaven 12
7100 Vejle

Trial protocol – english translation

Introduction

The purpose of the trial is to observe effects of an intervention consisting of 60 minutes of physical activity on all school days on cognition and academic performance in students attending 6th and 7th grade. Main outcome will be executive function which refers to the ability to modulate targeted actions in response to surroundings. Executive functions are divided into three sub-categories: working memory, self-control and cognitive flexibility (1). Early studies have clearly demonstrated an association between physical fitness and cognitive abilities in children (2). It has also been found that executive function is a predictor of mathematical as well as reading competences in every school year (3). Hence, our hypothesis is that an increased physical activity level can improve executive functions and academic performance in students attending 6th and 7th grade.

Data from the first study in learning, cognition and movement in Denmark has just been completed. The study, which involves 600 1st grade children and is a part of the CHAMPS-study DK (4), is investigating effects of physically active mathematics teaching og six hours of weekly physical education on executive functions, creative abilities and academic performance. To gain a complete picture of the relations between physical activity and cognitive performance in schoolchildren there is a need for studies including older student's as well.

Background

A few large meta-analyses on physical activity and cognitive outcomes have been performed (2, 5, 6). Both the study by Fedewa & Ahn and Sibley and colleagues reports an overall effect size of 0.32 (5, 6). The most recent analysis, which only addresses executive functions, reports only a significant effect of acute physical activity while it finds no effect of more long-term exercise (2). A number of reviews on the association between physical activity and academic performance have been performed (5, 7-11). Three of these reports a positive association (5, 7, 10), two concludes that a positive or no relation exist and that it will therefore be without risk of decreased academic performance to increase amounts of physical activity provided in schools (8, 11). An earlier review concludes that more studies are needed (9). The available evidence thus suggests that it is possible to improve both cognitive and academic performance by increasing activity levels. The present study aims to specifically investigate how such an intervention can be implemented in the Danish public school, which results it will provide and how the intervention is received by the school staff as well as students.

Ethics committee of Southern Denmark
Regionshuset, Damhaven 12
7100 Vejle

Methods, design and subjects

The trial design is randomized and controlled with randomization performed at the school level. Schools are randomized to either intervention or comparison by draws. Randomization will be performed after consent has been given from schools. Intervention schools will receive a “package” of activity possibilities which will enable students to gain 60 minutes of physical activity on each school day during the intervention period. The package is primarily targeted activities in school or to and from school (physically active teaching, power breaks, games and sports during recess, active transportation to and from school). Control schools will after the intervention and final collection of data receive the same package as the intervention.

Time flow	
October 2013	Randomization of schools to intervention or control status
October 2013	Meetings at schools with parents/teachers regarding the project
November-December 2013	Development of competences for intervention school teacher
November-December 2013	Baseline assessments
January 2014	Start intervention
May 2014	Endline assessment + end of intervention
August-September 2014	Development of competences for control school teacher

To ensure sufficient statistical power to observe an effect of the intervention we performed a power calculation. This took the clustering of students within schools into account by assuming a school level cluster-effect of 2%. Based on this, 17 schools needs to be included with approximately 40 participating students aged 12-14 years, thus 680 students are required to achieve standard levels of power. A cluster-effect refers to the fact that students will influence on each other in a similar environment, meaning that student in the same school will be more similar than students in different schools. The criterion for inclusion in the trial is that the student should be enrolled in a Danish 6th or 7th grade and follow normal curriculum in mathematics. Private schools or student receiving other mathematics-teaching than class-based teaching (e.g. one-to-one teaching, special-teaching or additional support during class) are excluded. No compensation for participation will be provided.

Ethical concerns

No clear risks, side effects or disadvantages are associated with the present trial. An indirect measure of maximal oxygen consumption will however be performed by an intermittent running test (Andersen test). This may cause exertion of the participant and in rare cases results in nausea, vomiting or dizziness. These side effects are however very rarely experienced and often coincide with general discomfort prior to the test or a lack of hydration. It will therefore be emphasized that the participant should only perform the test if the participant is feeling healthy and capable of performing the physical exertion. In case of circulatory issues or diseases a specific judgement about participation in the test will be made in agreement with the participant’s parents or legal guardian. Participants are covered by “Patientforsikringen” with respect to the “Lov om Klage- og

Erstatningsadgang" within the health system. In case of any damages that the University of Southern Denmark (SDU) may be liable of according to the "Patientforsikringen", these expenses will be covered by SDU. The trial has been filed with the "Regionernes fællesanmelse" and the Danish Data Protection Agency. All data, including personal information, will be handled confidentially in referral to "Persondataloven". Participants are in their full right to withdraw their consent and leave the trial. Participants are anonymous and exclusively identified by a code number. The trial may prove beneficial not only for participating students and schools but for schoolchildren in all of Denmark as well, as the trial can demonstrate the importance of increased movement in and outside of teaching. The limited risks and side effects are thus outweighed by the benefits.

Economy and publication of data

The trial is sponsored and initiated by "Ministeriet for Børn og Undervisning" (now Ministry of Education) which has granted 5.147.00 DKK to the project "Forsøg med Læring i Bevægelse" where LCoMotion is a part. Payment will take place in three rates. No affiliation with the sponsor exists. Results of the trial, positive as well as negative or inconclusive, will be published in a rapport to the Ministry of Education and in scientific journals.

Contact

First contact to schools was June where schools received oral as well as written information on the trial via electronic mail or letters. Oral information was given by post.doc., Anna Bugge, who have a short presentation for all invited schools. The written contact was initiated by research assistant Sidsel Louise Domazet. It is expected that schools will give their final decision on participation no later than 31th of August 2013. During fall 2013, meetings for school-leaders and participating staff will be held at schools where the research team will also be present. As such, a meeting will be hold at each school during fall 2013 where parents/guardians are invited to hear about the trial. After this, 6th and 7th grade students and their parents on participating schools will receive an information letter in which they are invited to join the study. As participants are minors, their parents or legal guardians will provide informed consent. Parents or legal guardians will be given opportunity to receive oral information on the content of the trial as well as consequences for their child. Consent must be returned to the research team prior to baseline assessments. It is expected that curses for development of teacher and student competences will be carried out in November 2013. Baseline assessments will take place in December with the intervention beginning in January 2014. Endline assessments will take place in May 2014 in close proximity to completion of the intervention.

Included tests

A test round will take place prior to and in the end of the intervention (baseline/endline). All tests will take place at individual schools with participants meeting at normal hours and tested during their scheduled school day. Additionally, SMS-tracking will continuously monitor the degree of implementation at the school, class and individual level.

Height

Height will be measured with a stadiometer with participants barefooted. Measurements to the nearest 0.5 cm will be made.

Weight

Weighting will be made on a floor-based scale with participants not wearing shoes or large jackets/shirts. Measurements to the nearest 0.1 cm will be made

Waist-circumference

Waist-circumference will be measured at the mid-point between the lower ribs and the iliac crest using a flexible tape. This is usually lined with the navel unless the participant is severely overweight. The measurement will be made on the skin by the end of a light exhale. The measurement will be performed twice and once more in case of a difference of more than 1 cm. Measurements will be made to the nearest 0.5 cm.

Hip-circumference

Hip-circumference will be measured at the widest position of the hip using a flexible tape. This is usually at trochanter major. The measurement will be made on the skin or light underwear. The measurement will be performed twice and once more in case of a difference of more than 1 cm. Measurements will be made to the nearest 0.5 cm.

Self-rated pubertal development

Participants will subjectively assess their sexual maturity based on a schema with pictures and text of secondary sex characteristics (5 stages according to Tanner). Breast development and pubic hair data will be collected in girls and genitalia and pubic hair in boys. Girls will further be asked to cross if they have had their period. Trained staff will inform the participant on the consent of the schema but the participant will be left alone for filling out the schema.

Physical activity

The physical activity level will be estimated by accelerometri (Actigraph GT3X og GT3X+) combined with information on cycling and swimming from questionnaires. Participants will wear the accelerometer for a minimum of 8 days and is encouraged to wear it during all waken hours. The accelerometer is not water resistant and must be taken of during shower, swimming or other water based activities. The

accelerometer can register motion in three axis (vertical-, sagittal-, transversalplane), measures 4,6cm x 3,3cm x 1,5cm and is carried at the hip using an elastic ban.

Cognition (executive functions)

Executive function will be assessed using a computer-based reaction test, Flanker Fish Task. This test uncovers the participants' ability to remember, inhibit and shift between different signals/tasks. On the computer screen a line of fish with arrows will appear which mark the respective fish's direction of swimming. The fish will point in a random direction, however, the four outer fish will always point in the same direction. During the first part of the test the participant is instructed to focus on the blue fish in the middle and press the arrow-key which marks the direction of the fish. In the second part, the participant will focus on the four outer pink fish and press the arrow-key corresponding to their direction. The third part of the test is a combination of the above such that dependent on the color of the fish being blue or pink the participant must focus on the middle fish or the outer fish. The test is adapted to the age group.

Fitness

Maximal oxygen consumption is measured indirectly by an intermittent running test (Andersen testen). Participants will alternate between 15 seconds of running and brake for a total of 10 minutes. Participants will prior to the test be informed that the purpose of the test is to travel as long a distance as possible. The track is 20 meters wide and market with cones. Students will touch the line of each end when they change directions. The test will be formed in two sections where participants will alternate between running and counting lanes for another student.

Mathematics test

Mathematic skills will be assessed by a standardized mathematics test developed for the 6th and 7th grade. The test will be of identical difficulty at both baseline and endline, will contain the same type of assignments but will not be identical.

Questionnaire

Participants as well as their parents or legal guardian will receive a paper based questionnaire which will attempt to uncover socioeconomic status (i.e. marital status, income, education, work, living), transportation to and from school, health habits and participation in sports. The questionnaire to participants will be distributed along with the accelerometer.

Ethics committee of Southern Denmark
Regionshuset, Damhaven 12
7100 Vejle

SMS-track

SMS track is intended for the weekly investigation of whether participants adhere to the recommended 60 minutes of physical activity on each day in and outside of class teaching. Further, it will be assessed how often teachers initiate active teaching during class or movement during school time. The SMS will be constructed to individually inquire on the amount and intensity of physical activity within a certain time period (e.g. how many minutes of physical activity did you participate in during recess during the last week?).

Interviews and video recordings

Two select schools will be further followed by a group of qualitative researchers who will use video recordings and interviews to investigate the implementation and perception of the intervention

Literature

1. Miyake A, Friedman NP, Emerson MJ, Witzki AH, Howerter A, Wager TD. The unity and diversity of executive functions and their contributions to complex "Frontal Lobe" tasks: a latent variable analysis. Cogn Psychol. 2000;41(1):49-100.
2. Verburgh L, Konigs M, Scherder EJ, Oosterlaan J. Physical exercise and executive functions in preadolescent children, adolescents and young adults: a meta-analysis. British journal of sports medicine. 2014;48(12):973-9.
3. Gathercole SE, Pickering SJ, Knight C, Stegmann Z. Working memory skills and educational attainment: Evidence from national curriculum assessments at 7 and 14 years of age. Applied Cognitive Psychology. 2004;18(1):1-16.
4. Wedderkopp N, Jespersen E, Franz C, Klakk H, Heidemann M, Christiansen C, et al. Study protocol. The Childhood Health, Activity, and Motor Performance School Study Denmark (The CHAMPS-study DK). BMC pediatrics. 2012;12:128.
5. Fedewa AL, Ahn S. The effects of physical activity and physical fitness on children's achievement and cognitive outcomes: a meta-analysis. Research quarterly for exercise and sport. 2011;82(3):521-35.
6. Sibley BA, Etnier JL. The relationship between physical activity and cognition in children: A meta-analysis. Pediatric exercise science. 2003;15(3):243-56.
7. Tomporowski PD, Davis CL, Miller PH, Naglieri JA. Exercise and Children's Intelligence, Cognition, and Academic Achievement. Educational psychology review. 2008;20(2):111-31.
8. Trudeau F, Shephard RJ. Physical education, school physical activity, school sports and academic performance. The international journal of behavioral nutrition and physical activity. 2008;5:10.
9. Taras H. Physical activity and student performance at school. Journal of School Health. 2005;75(6):214-8.
10. Singh A, Uijtdewilligen L, Twisk JWR, van Mechelen W, Chinapaw MJM. Physical Activity and Performance at School A Systematic Review of the Literature Including a Methodological Quality Assessment. Arch Pediat Adol Med. 2012;166(1):49-55.
11. CDC. Centers for Disease Control and Prevention. The Association between school based physical activity, including physical education, and academic performance. 1-84 2010 Atlanta, GA: US, Department of Health and Human Services.
