# Supplementary material for: Effectiveness of a School-Based Physical Activity Intervention on Cognitive Performance in Danish Adolescents: LCoMotion—Learning, Cognition and Motion – A Cluster Randomized Controlled Trial
Source: PLoS One. 2016 Jun 24;11(6):e0158087. doi: 10.1371/journal.pone.0158087 (PMC4920412; doi:10.1371/journal.pone.0158087)
Supplement: S3 File — Approved trial protocol (Danish). (PDF) [file pone.0158087.s003.pdf]

Til Den Videnskabsetiske Komité for Region Syddanmark
Regionshuset, Damhaven 12
7100 Vejle

C. Forsøgsprotokollen

Introduktion

Forsøgets formål er at observere effekten af indførelse af 60 minutters fysisk aktivitet på alle skoledage på kognition og akademisk formåen hos elever i 6. og 7. klasse. Hoved ”outcomes” vil være eksekutive funktioner, der refererer til evnen til at regulere igangværende, målrettet adfærd i forhold til omstændighederne. De inddeles i følgende tre underkategorier: arbejdshukommelse, selvkontrol og kognitiv fleksibilitet (1). Tidligere studier har vist en klar sammenhæng mellem fysisk form og kognitive evner hos børn (2). Samtidig er det fundet at eksekutive funktioner kan forudsige matematiske færdigheder samt læsekompetence i samtlige skoleår (3). Vores hypotese er derfor, at et øget fysisk aktivitetsniveau kan forbedre de eksekutive funktioner og fremme den faglige præstation i de boglige fag hos elever på 6. og 7. klassetrin.
Data fra det første studie omhandlende læring, kognition og bevægelse i Danmark er netop færdigindsamlet. Studiet, som involverer 600 børn i første klasse og er en del af Svendborg Projektet, The CHAMPS study-DK (4), undersøger effekten af aktiv matematikundervisning og 6 timers ugentlig idrætsundervisning på eksekutive funktioner, kreative evner og akademisk kunnen. For at opnå et fyldestgørende billede af sammenhængen mellem fysisk aktivitet og kognitive færdigheder hos skolebørn, er der behov for studier, der inddrager elever fra folkeskolens mellemtrin og udskoling.

Baggrund

Flere større meta-analyser er blevet udført vedrørende fysisk aktivitets effekt på kognitive outcomes (2,5,6). Både studiet af Fedewa & Ahn, samt Sibley og kollegaer finder en overordnet effektstørrelse på 0.32 (5,6). Den nyeste analyse, der alene kigger på eksekutive funktioner, finder kun en signifikant effekt af akut fysisk aktivitet, mens der ikke er signifikant effekt af længerevarende træning (2). Der er også lavet et antal reviews om sammenhængen mellem fysisk aktivitet og akademisk formåen f.eks. (5,7,8,9,10,11). Tre af disse finder en positiv association (5,7,10), to konkludere at der enten er en positiv eller ingen sammenhæng og at man derfor uden risiko for nedsat akademisk performance kan forøge mængden af fysisk aktivitet i skolen (8,11), mens et tidligt review konkluderer, at der endnu mangler viden på området (9). Der er således meget der tyder på, at man kan forbedre både kognitive funktioner og akademisk formåen, hvis man kan øge det fysiske aktivitetsniveau. Indeværende studie sigter mod specifikt at undersøge hvordan en sådan intervention kan implementeres i den danske folkeskole, hvilke resultater det vil give, og hvordan interventionen modtages af skolepersonale, samt ikke mindst, af eleverne i 6. og 7. klasse.

Til Den Videnskabsetiske Komité for Region Syddanmark
Regionshuset, Damhaven 12
7100 Vejle

Metode, design og forsøgspersoner

Forsøgets anvendte design er et randomiseret kontrolleret forsøgsdesign, hvor randomiseringen foregår på skoleniveau. Skolerne randomiseres til hhv. interventions- og sammenligningsskoler ved lodtrækning. Randomiseringen finder sted efter endeligt tilsagn om deltagelse fra de enkelte skoler. Interventionsgruppen vil modtage en samlet pakke af aktivitetstilbud, som skal gøre det muligt for eleverne at imødekomme anbefalingen om 60 minutters daglig fysisk aktivitet i interventionsperioden. Aktivitetstilbuddene er primært rettet mod aktiviteter, der foregår i eller til og fra skole (fx aktiv undervisning, power breaks, leg og sport i frikvarterer, aktiv transport til og fra skole). Kontrolgruppen vil efter interventionsperiodens ophør og når alle data er indsamlede modtage samme aktivitetstilbud som interventionsgruppen.

**Tidsplan**	
oktober 2013	Lodtrækning i forbindelse med fordeling af interventions- og sammenligningsskoler
oktober 2013	Møde på skolerne for forældre/værge om projektet
november-december 2013	Kompetenceudvikling af lærere og elever på interventionsskoler
november-december 2013	Første testrunde (baseline)
januar 2014	Interventionen starter
maj 2014	Anden testrunde (endline) og interventionen slutter
August-september 2014	Kompetenceudvikling af lærere og elever på sammenligningsskoler

For at opnå tilstrækkelig statistisk styrke til at observere en effekt af interventionen, er der foretaget power beregninger, som tager højde for en clustereffekt svarende til 0.02 (2 %) på skoleniveau. Ud fra disse beregninger skal der inkluderes 17 skoler i forsøget. Hvis vi yderligere antager at hver skole bidrager med ca. 40 elever i alderen 12-14 år, svarer det endelige antal af forsøgspersoner til 680. En clustereffekt refererer til den afsmittende adfærd, der opstår mellem individer under samme omstændigheder og miljømæssig påvirkning, forstået således at der er en større sammenhørighed i adfærd hos elever fra samme skole end hos elever fra forskellige skoler. Inklusionskriterierne for at deltage i forsøget er, at forsøgspersonen skal være indskrevet i en dansk 6. eller 7. folkeskoleklasse og følge den obligatoriske klasseundervisning i matematik. Privatskoler og elever, der modtager anden matematikundervisning end den klasseordinerede undervisning (fx én-til-én undervisning, specialklasse undervisning, støttepædagog) er ekskluderet fra forsøget. Der gives ikke vederlag til forsøgspersonerne for deltagelse.

Til Den Videnskabsetiske Komité for Region Syddanmark
Regionshuset, Damhaven 12
7100 Vejle

Etiske overvejelser

Der er ingen umiddelbare risici, bivirkninger eller ulemper forbundet med nærværende forsøg. Der udføres dog en indirekte måling af den maksimale iltoptagelse ved en interval løbetest (Andersen testen), der kan forårsage fysisk udmattelse af forsøgsdeltageren og i sjældne tilfælde resultere i kvalme, opkast eller svimmelhed. Disse bivirkninger opleves dog meget sjældent, og opstår oftest pga. generel utilpashed før løbet, mangel på væske eller stor mæthed forud for løbet. Der lægges derfor vægt på, at forsøgsdeltageren kun løber, hvis vedkommende føler sig rask og i stand til at klare den fysiske belastning. Desuden spørges der på forhånd ind til hjerteproblemer hos forsøgspersonen. Eventuelle kredsløbsmæssige problemer eller lidelser vurderes i hvert enkelt tilfælde, og der tages i samråd med forældre/værge stilling til, hvorvidt forsøgspersonen kan deltage i løbetesten.

Forsøgspersonerne er dækket af Patientforsikringen i henhold til Lov om Klage- og Erstatningsadgang indenfor sundhedsvæsenet. Eventuelle skader som SDU må kunne blive ansvarlig for ved regreskrav fra Patientforsikringen dækkes af SDU selv, idet SDU er en selvforsikrende institution.

Forsøget er anmeldt til regionernes fællesanmeldelse til Datatilsynet. Alle data herunder personoplysninger behandles fortroligt i henhold til persondataloven. Forsøgspersonen er i sin fulde ret til at annullere samtykket og forlade forsøget. Forsøgspersonerne er anonyme og er udelukkende identificeret ved et kodenummer. Projektet kan vise sig yderst gavnligt; ikke kun for medvirkende elever og skoler, men for skolebørn i hele Danmark, da forsøget kan tydeliggøre vigtigheden af øget bevægelse i og udenfor undervisningen. De begrænsede risici og bivirkninger ved forsøget opvejes derfor klart af forsøgets fordele.

Økonomi og offentliggørelse af data

Forsøget er finansieret og initieret af Ministeriet for Børn og Undervisning, som har bevilliget 5.147.000 kr. til projekt ”Forsøg med læring i bevægelse” hvor projekt LCoMotion indgår. Udbetalingen sker i tre rater. Forsøgsansvarlige har ingen tidligere tilknytning til støttegiver.

Resultaterne af forsøget, såvel positive, negative som inkonklusive fund, vil blive offentliggjort dels i en rapport til Ministeriet for Børn og Undervisning og dels ved publicering af videnskabelige artikler i relevante videnskabelige tidsskrifter.

Til Den Videnskabsetiske Komité for Region Syddanmark
Regionshuset, Damhaven 12
7100 Vejle

Kontakt

Den første kontakt til skolerne fandt sted i juni, hvor skolerne modtog mundtlig samt skriftlig information om forsøget via elektronisk post og brevpost. Den mundtlige information blev givet af post.doc, Anna Bugge, som holdt et kort oplæg for samtlige inviterede skoler. Den skriftlige kontakt blev indledt af videnskabelig assistent, Sidsel Louise Domazet. Det forventes at skolerne melder endeligt tilbage senest d. 31. august 2013, om de ønsker at deltage i forsøget. Der vil i løbet af efteråret 2013 blive afholdt et møde for skoleledelsen og medvirkende medarbejdere på skolerne, hvor forskergruppen vil være repræsenteret. Der vil ligeledes blive afholdt et møde på hver enkel skole i efteråret 2013, hvor forældre/værge er indbudt til at høre nærmere om forskningsprojektet. Herefter vil eleverne i 6. og 7. klasse på de deltagende skoler samt deres forældre/værge modtage et informationsbrev, hvor de inviteres til at deltage i forsøget. Da forsøgspersonerne er mindreårige, indhentes der stedfortrædende samtykke fra begge indehavere af forældremyndigheden. Forældre/værge har mulighed for at få mundtlig uddybelse af forskningsprojektets indhold samt konsekvenser for deres barn. Samtykkeerklæringen skal returneres til forskergruppen inden afviklingen af første testrunde. Det forventes at uddannelseskurser til lærere og elever afvikles i slutningen af efteråret (november 2013). Første testrunde (baseline) vil foregå i december 2013, hvor selve interventionen starter op efter juleferien i januar 2014. Anden testrunde (endline) vil foregå i maj 2014 i umiddelbar nærhed af interventionens ophør.

Testbatteri

Der vil være en testrunde forud for og i slutningen af interventionen (baseline/endline). Alle tests vil foregå på de enkelte skoler, hvor forsøgsdeltagerne møder til normal tid og testes i løbet af deres skemalagte skoletid. Derudover vil der blive foretaget tests (SMS Track) kontinuerligt i interventionsperioden, som skal afdække graden af implementering af interventionen både på skole-, klasse- og individniveau.

Højde

Højdemålingen foretages med et stadiometer, der placeres stabilt op af en væg. Målingen udføres uden sko. Forsøgspersonens ryg og hæle rør bagkanten af stadiometret let uden at læne sig op af dette. Der måles til nærmeste 0,5 cm.

Vægt

Vejningen foretages med en gulvvægt. Målingen udføres uden sko og store jakker/bluser. Der måles til nærmeste 0,1 kg.

Taljeomkreds

Taljevidden måles med et målebånd på det midterste punkt i linjen mellem den nederste ribbenskant og hoftekammen (crista iliaca). Dette punkt er som oftest ud for navlen, såfremt forsøgspersonen ikke er kraftigt overvægtig. Målingen foretages på bar hud i slutningen af en let udånding. Målingen foretages to gange og gentages yderligere, hvis de to målinger divergerer mere end 1 cm. Der måles til nærmeste 0,5 cm.

Hofteomkreds

Hoftevidden måles med et målebånd på det bredeste sted i hofteregionen. Dette punkt er som oftest ud for hoftebenet (trochanter major). Målingen foretages på bar hud eller på undertøj. Målingen foretages to gange og gentages yderligere, hvis de to målinger divergerer mere end 1 cm. Der måles til nærmeste 0,5 cm.

Selvvurderet pubertetsbestemmelse

Forsøgspersonen vurderer ud fra et skema med tekst og billeder (Tanner stages), hvor vedkommende er i sin udvikling af sekundære kønstræk herunder udvikling af bryster samt kønsbehåring hos piger og udvikling af genitalier samt kønsbehåring hos drenge. Derudover afkrydser pigerne, om de har fået menstruation. Skemaet gennemgås af den forsøgsansvarlige, så forståelsen sikres, hvorefter forsøgspersonen selv afkrydser skemaet uden indblanding af forsøgsansvarlige.

Fysisk aktivitet

Det fysiske aktivitetsniveau estimeres ud fra brug af accelerometri (Actigraph GT3X og GT3X+) kombineret med oplysninger om cykling og svømning fra spørgeskema. Forsøgspersonen skal gå med accelerometret i minimum 8 dage og opfordres til at bære det alle døgnets vågne timer. Accelerometret tåler ikke vand og skal derfor afmonteres i forbindelse med bad, svømning eller anden vandaktivitet. Accelerometret kan registrere bevægelse i tre akser (vertikal-, sagittal-, transversalplan), måler 4,6cm x 3,3cm x 1,5cm og placeres på hoften med et elastikbælte.

Kognition (eksekutive funktioner)

De eksekutive funktioner undersøges ved en computerbaseret reaktionstest, Flanker Fish Task. Denne test afdækker forsøgspersonens evne til at kunne huske, inhibere samt skifte mellem

forskellige signaler/opgaver. På computerskærmen vises der en række af fisk med pile, der markerer den enkelte fisks svømmeretning. Fiskene peger i uvilkårlig retning, men er dog organiseret således, at de fire yderste fisk altid peger i samme retning. I den første del af testen er det forsøgspersonens opgave at fokusere på den blå fisk i midten og trykke på den piltast på tastaturet, der markerer fiskens svømmeretning. I testens anden del har forsøgspersonen til opgave at fokusere på de fire yderste lyserøde fisk og markere deres svømmeretning ved at bruge piltasterne på tastaturet. Den tredje del af testen kombinerer de to foregående opgaver, således at alt efter om fisken er blå eller lyserød, skal forsøgspersonen fokusere på den midterste eller de yderste fisk og angive svømmeretningen. Testen er tilpasset aldersgruppen i hastighed, hvormed signalerne optræder.

Fitness

Den maksimale iltoptagelse måles indirekte ud fra en interval løbetest (Andersen testen), hvor forsøgspersonen løber 15 sekunder og holder pause i 15 sekunder. Testen varer 10 minutter, hvoraf 5 minutter er løb i et selvvalgt tempo og de resterende 5 minutter er pause. Forsøgspersonen får at vide forud for løbet, at meningen med testen er at løbe så langt og så hurtigt som muligt. Banen er 20 m lang (håndboldbanehalvdel) og markeret med kegler, hvor der vendes i hver ende ved at røre linjen mellem keglerne. Klassen opdeles i to, hvoraf den ene halvdel starter med at gennemføre løbetesten, mens den anden halvdel tæller antallet af gennemførte omgange (20 m), hvorefter de to halvdele bytter.

Matematiktest

Forsøgspersonens matematiske færdigheder afdækkes ved en standardiseret matematikprøve, der er udviklet specifikt til hhv. 6. og 7. klassetrin. Testen er af samme sværhedsgrad ved baseline og endline målingerne og indeholder de samme typer af opgaver dog uden at være identiske.

Spørgeskema (se bilag)

Både forsøgspersonen samt dennes forældre/værge modtager et spørgeskema i papirform, der søger at afdække familiens socioøkonomiske status (herunder; civilstand, husstandsindkomst, uddannelsesniveau, arbejdsforhold, leveforhold), transport til og fra skole, sundhedsvaner samt idrætsdeltagelse. Spørgeskemaet til eleverne udleveres sammen med accelerometret.

SMS Track

SMS Track er tiltænkt som en ugentlig opfølgning på, om eleverne deltager i de anbefalede 60 minutters daglig fysisk aktivitet i og uden for undervisningen, og om hvor ofte lærerne initierer

aktiv undervisning eller bevægelse i skoletiden. SMS'en er konstrueret således, at der sendes en besked til den medvirkendes mobiltelefon med spørgsmål om mængden samt intensiteten af fysisk aktivitet på et givet tidspunkt (fx hvor mange minutter har du været aktiv i frikvartererne i løbet af den forgangne uge).

Interview og videooptagelse

To udvalgte skoler vil desuden blive fulgt af en gruppe kvalitative forskere, der gennem interviews og videooptagelser undersøger implementeringen af interventionen og modtagelsen af denne.

Litteraturliste

1 Miyake A, Friedman NP, Emerson MJ, Witzki AH, Howerter A, Wager TD. The unity and diversity of executive functions and their contributions to complex "Frontal Lobe" tasks: a latent variable analysis. Cogn Psychol 2000 August;41(1):49-100.

2 Verburgh L, Konigs M, Scherder EJ, Oosterlaan J. Physical exercise and executive functions in preadolescent children, adolescents and young adults: a meta-analysis. Br J Sports Med 2013 March 6.

3 Gathercole SE, Pickering SJ, Knight C, Stegmann Z. Working memory skills and educational attainment: evidence from National Curriculum assessments at 7 and 14 years of age. Appl.Cogn.Psychol. 18, 1-16. 2004.

4 Wedderkopp N, Jespersen E, Franz C, Klakk H, Heidemann M, Christiansen C, Moller NC, Leboeuf-Yde C. Study protocol. The Childhood Health, Activity, and Motor Performance School Study Denmark (The CHAMPS-study DK). BMC Pediatr 2012;12:128.

5 Fedewa AL, Ahn S. The effects of physical activity and physical fitness on children's achievement and cognitive outcomes: a meta-analysis. Res Q Exerc Sport 2011 September;82(3):521-35.

6 Sibley BA, Etnier JL. The relationship between physical activity and cognition in children: A meta-analysis. Pediatr.Exerc.Sci. 15, 243-256. 2003.

7 Tomporowski PD, Davis CL, Miller PH, Naglieri JA. Exercise and Children's Intelligence, Cognition, and Academic Achievement. Educ Psychol Rev 2008 June 1;20(2):111-31.

8 Trudeau F, Shephard RJ. Physical education, school physical activity, school sports and academic performance. Int J Behav Nutr Phys Act 2008;5:10.

9 Taras H. Physical activity and student performance at school. J Sch Health 2005 August;75(6):214-8.

10 Singh A, Uijtdewilligen L, Twisk JW, van MW, Chinapaw MJ. Physical activity and performance at school: a systematic review of the literature including a methodological quality assessment. Arch Pediatr Adolesc Med 2012 January;166(1):49-55.

11 CDC. Centers for Disease Control and Prevention. The Association between school based physical activity, including physical education, and academic performance. 1-84. 2010. Atlanta, GA: U.S., Department of Health and Human Services.
