# Supplementary material for: Effectiveness of a School-Based Physical Activity Intervention on Cognitive Performance in Danish Adolescents: LCoMotion—Learning, Cognition and Motion – A Cluster Randomized Controlled Trial
Source: PLoS One. 2016 Jun 24;11(6):e0158087. doi: 10.1371/journal.pone.0158087 (PMC4920412; doi:10.1371/journal.pone.0158087)
Supplement: S1 Table — Test-retest reliability of the flanker task and mathematics skills. (DOCX) [file pone.0158087.s005.docx]

**S1 Table. Reliability measures.** Test-retest reliability of the flanker task and mathematics skills

The test- retest reliability of the flanker task and the mathematics performance test was assessed 14 days apart in four classes (2 from 6^th^ grade and 2 from 7^th^ grade). These classes were from a school not participating in the trial. Results are presented in table S1.1. Response accuracy is presented as median (inter-quartile range) and reaction time and mathematics score as mean (standard deviation).

Table S1.1. Fourteen days test-retest reliability of the flanker task and mathematics performance

|  | Test 1 | Test 2 | Difference | Spearman’s rho | 95% CI of rho | Spearman’s rho  p-value |
| --- | --- | --- | --- | --- | --- | --- |
| **Accuracy (%)** | **Median (IQR)** | **Median (IQR)** | **Median (IQR)** |  |  |  |
| Congruent | 97.9 (92.6-100) | 98.6 (96.0-100) | 0.1 (-0.7 – 3.4) | 0.45 | 0.22, 0.63 | < 0.001 |
| Incongruent | 81.9 (73.5 -92.1) | 90.6 (82.4-95.9) | 3.9 (-1.8 – 11.4) | 0.63 | 0.45, 0,76 | < 0.001 |
| Interference score | 11.5 (6.5 – 20.3) | 7.4 (4.0 – 11.8) | - 2.8 (-9.8 – 2.1) | 0.46 | 0.23, 0.64 | < 0.001 |
| **RT (ms)** | **Mean (SD)** | **Mean (SD)** | **Mean (SD)** |  |  |  |
| Congruent | 453.3 (77.3) | 455.9 (78.2) | 2.6 (46.9) | 0.84 | 0.74, 0.90 | < 0.001 |
| Incongruent | 544.5 (111.7) | 525.7 (93.0) | -18.8 (59.3) | 0.84 | 0.74, 0.90 | < 0.001 |
| Interference score | 91.2 (50.2) | 69.8 (35.8) | - 21.4 (33.7) | 0.68 | 0.51, 0,80 | < 0.001 |
| **Mathematics score** | **Mean (SD)** | **Mean (SD)** | **Mean (SD)** |  |  |  |
|  | 22.9 (9.8) | 23.4 (9.6) | 0,5 (4.0) | 0.92 | 0.86 – 0.95 | < 0.001 |

*RT: reaction time. RT and mathematics score are displayed as mean and standard deviations (SD) and accuracy as median and inter-quartile range (IQR). 60 participants completed the Flanker task at both time points and 66 completed the mathematics test at both time points.* *Mathematics score can range from 0-50 points*
