# Supplementary material for: Effectiveness of a School-Based Physical Activity Intervention on Cognitive Performance in Danish Adolescents: LCoMotion—Learning, Cognition and Motion – A Cluster Randomized Controlled Trial
Source: PLoS One. 2016 Jun 24;11(6):e0158087. doi: 10.1371/journal.pone.0158087 (PMC4920412; doi:10.1371/journal.pone.0158087)
Supplement: S2 Table — Results of multiple imputation analyses. (DOCX) [file pone.0158087.s006.docx]

**S2 Table.** **Multiple imputation analyses**. Results of multiple imputation analyses.

Imputation of variables was performed by using chained equations (“mi impute chained”) in Stata v13.1. Beta coefficients and standard errors were obtained based on 20 imputed datasets. At follow up, 21 (10%) students at intervention schools, and 52 (11%) students at control schools who obtained a baseline measure did not obtain data. The primary reason was absence from school. All students with an observed baseline value of the respective outcome were included in the intention to treat analyses (“mi estimate”). The number of Imputed values ranged from 81 (mathematics test) to 129 (cardiorespiratory fitness test) which corresponds to 12% and 22% of the observations, respectively. Imputation models were visually checked for convergence and the reproducibility of the estimates where inspected by Monte Carlo errors. The imputation analyses are based on the assumption of data being missing at random conditional on the observed covariates (missing at random).

Imputation was performed for outcome variables, baseline variables (log transformed if skewed) and indicators of socioeconomic status and pubertal development. Also included in the imputation model were gender, school year (grade 6 or 7) and dummy indicators for school and classes (as fixed effects). Imputation was done separately for the intervention and the control group. Ethnicity was not included in the imputation model due to problems with perfect prediction which could not be overcome by using the “augment” option. Results are showed in table S2.1.

Table S2.1. Intervention effects on primary and secondary outcomes from multiple imputation analyses.

| Outcome |  |  | Adjusted^#^ difference in change  (beta*) | 95%CI | p-value |
| --- | --- | --- | --- | --- | --- |
|  | Intervention  n= | Control  n= |  |  |  |
| Accuracy congruent  (%) | 198 | 460 | -0.4 | -1.1 – 0.4 | 0.32 |
| Accuracy incongruent  (%) | 198 | 460 | 0.9 | -0.6 – 2.4 | 0.25 |
| RT congruent  (ms) | 198 | 460 | 2.1 | -5.6 – 9.8 | 0.59 |
| RT incongruent  (ms) | 198 | 460 | 6.5 | -2.1 – 15.1 | 0.14 |
| Reaction time interference score (ms) | 198 | 460 | 5.2 | 0.6 – 9.8 | 0.03 |
| Accuracy interference score (%) | 198 | 460 | 1.1 | -0.2 - 2.4 | 0.10 |
| Mathematics  Test^¤^ | 215 | 476 | -0.2 | -1.7 - 1.2 | 0.74 |
| Cardiorespiratory fitness (distance, m)^*^ | 202 | 416 | 8.2 | -5.8 - 22.2 | 0.25 |
| Waist circumference^^^ | 211 | 477 | 0.7 | -0.7 - 2.2 | 0.31 |
| BMI | 212 | 478 | -0.1 | -0.2 - 0.0 | 0.17 |

^#^Adjusted for baseline value, gender and grade level

*Beta: Intervention=1.

^¤^further adjusted for class cluster as a random effect

^*^further adjusted for overweight status at baseline

^^^further adjusted for school cluster as a random effect

Values are mean (SD) unless stated otherwise. Mathematics performance can range from 0-50 points. RT: reaction time, CI: confidence interval, BMI: body mass index.
